# Identifying a lactic acid metabolism-related gene signature contributes to predicting prognosis, immunotherapy efficacy, and tumor microenvironment of lung adenocarcinoma

**DOI:** 10.3389/fimmu.2022.980508

**Published:** 2022-10-07

**Authors:** Fangchao Zhao, Zengying Wang, Zhirong Li, Shiquan Liu, Shujun Li

**Affiliations:** ^1^ Department of Thoracic Surgery, The Second Hospital of Hebei Medical University, Shijiazhuang, China; ^2^ Graduate School of Hebei Medical University, Shijiazhuang, China; ^3^ Department of Ophthalmology, The Third Hospital of Hebei Medical University, Shijiazhuang, China; ^4^ Clinical Laboratory Center, The Second Hospital of Hebei Medical University, Shijiazhuang, China; ^5^ Department of Thoracic Surgery, Affiliated Hospital of Chengde Medical University, Chengde, China

**Keywords:** lung adenocarcinoma, lactic acid metabolism, pyroptosis, tumor microenvironment, immunotherapy

## Abstract

Lactic acid, once considered as an endpoint or a waste metabolite of glycolysis, has emerged as a major regulator of cancer development, maintenance, and progression. However, studies about lactic acid metabolism-related genes (LRGs) in lung adenocarcinoma (LUAD) remain unclear. Two distinct molecular subtypes were identified on basis of 24 LRGs and found the significant enrichment of subtype A in metabolism-related pathways and had better overall survival (OS). Subsequently, a prognostic signature based on 5 OS-related LRGs was generated using Lasso Cox hazards regression analysis in TCGA dataset and was validated in two external cohorts. Then, a highly accurate nomogram was cosntructed to improve the clinical application of the LRG_score. By further analyzing the LRG_score, higher immune score and lower stromal score were found in the low LRG_score group, which presented a better prognosis. Patients with low LRG_score also exhibited lower somatic mutation rate, tumor mutation burden (TMB), and cancer stem cell (CSC) index. Three more independent cohorts (GSE126044: anti-PD-1, GSE135222: anti-PD-1, and IMvigor210: anti-PD-L1) were analyzed, and the results showed that patients in the low LRG_score category were more responsive to anti-PD-1/PD-L1 medication and had longer survival times. It was also determined that gefitinib, etoposide, erlotinib, and gemcitabine were more sensitive to the low LRG_score group. Finally, we validated the stability and reliability of LRG_score in cell lines, clinical tissue samples and HPA databases. Overall, the LRG_score may improve prognostic information and provide directions for current research on drug treatment strategies for LUAD patients.

## Introduction

In 2020, 2.2 million new cases of lung cancer and 1.8 million deaths appeared worldwide, ranking 2nd and 1st in incidence and mortality of malignant tumors, respectively (1, 2). The incidence and mortality of lung cancer in China occupy 37.0% and 39.8% of the world, and the 5-year survival rate is only 10% to 15% (3). Non-small cell lung cancer (NSCLC) occupies 85% of lung cancer cases, and lung adenocarcinoma (LUAD) is one of the major pathological types of NSCLC (4). Although breakthroughs have been made in the diagnosis, prognosis, and treatment of LUAD, the long-term survival rate is still low (5, 6). Therefore, further elucidation of the molecular mechanisms of LUAD progression will help to explore safety and effective biomarkers or therapeutic targets of lung cancer.

For a long time, the role of lactic acid in tumorigenesis and progression has been underestimated, primarily viewed as a metabolic waste produced by glycolysis or a marker of poor prognosis (7, 8). In recent years, lactic acid has been found to play a key role in tumor growth, immune escape, angiogenesis, invasion and metastasis, metabolic regulation, and tumor microenvironment (TME) cell-cell interactions (9, 10). For example, lactic acid produced by cancer cells is secreted into the extracellular environment and promotes cancer progression (11). Proton-coupled lactate efflux in cancer cells or stromal cells can regulate TME (including cell invasion, angiogenesis, survival signals, metastasis and development, and escape immune surveillance) to promote tumor progression (12). Extracellular acidosis inhibits T-cell-mediated immunity, and neutralizing tumor acidity may enhance the antitumor effect of immunotherapy (13). However, there is still a lack of bioinformatic research on lactic acid metabolism-related genes (LRGs) in LUAD.

This research assessed the expression profiles of LRGs in a comprehensive way. First, 535 patients with LUAD were divided into two subtypes on basis of LRG expression levels. The robust and effective prognostic signature (LRG_score) were constructed on basis of differentially expressed genes (DEGs) identified based on the two lactic acid metabolism subtypes. The prognostic LRG_score independently forecast the survival result of LUAD patients and effectively told apart LUAD patients sensitive to drug treatment. Besides, the association between the LRG_score and TME, mutation, tumor mutation burden (TMB), and cancer stem cell (CSC) index was investigated.

## Materials and methods

### Data source

The RNA-seq transcriptome data (FPKM) and related clinical information of LUAD patients were extracted from TCGA database (LUAD samples: 535, normal samples: 59). Additionally, 24 LRGs were retrieved from MSigDB database for subsequent bioinformatics analysis. The TCGA-LUAD as the training cohort in this study. The GSE31210 cohort and the GSE30219 cohort from the GEO database were obtained as two independent validation cohorts. To predict the immunotherapy response, GSE126044 and GSE135222 microarrays with treatment information from NSCLC patients receiving anti-PD-1 therapy were gathered. The IMvigor210 dataset contains 298 urothelial carcinoma patients who were treated with anti-PD-L1. The “limma” package was used to analyze differentially expressed LRGs between normal samples and tumor samples from TCGA. Online tools on the STRING website were employed to map a protein-protein interaction (PPI) network.

### Consensus clustering analysis of LRGs

Consensus unsupervised clustering analysis was made with the R “ConsensusClusterPlus” package to categorize patients into different molecular subtypes on basis of LRG expression levels. Subsequently, the relationship between molecular subtypes and clinicopathological features and prognosis was compared. Then, principal component analysis (PCA) was performed using the “ggplot2” R package. In addition, this paper compared the overall survival (OS) time between the two subtypes via Kaplan-Meier analysis. The proportions of 23 human immune cell subsets were calculated with the CIBERSORT algorithm. In addition, the gene set variation analysis (GSVA) of R software was used to assess differences in biological pathways between different subtypes.

### DEG identification and functional annotation

DEGs between the different lactic acid metabolism subtypes were identified using the “limma” package in R with a fold-change of 1 and an adjusted p-value of<0.05. To further explore the potential functions of lactic acid metabolism subtypes-related DEGs and identify the related gene functions and enriched pathways, functional enrichment analyses were executed on the DEGs using the “clusterprofiler” package in R.

### Construction of the prognostic LRG_score and nomogram

Using the “limma” R package (the filter standard is: LogFC > 1, and the corrected p value is< 0.05) and univariate Cox analysis, LRG-related genes with differential expression and prognostic value were identified in the TCGA cohort. Based on the expression of prognostic LRGs and survival data, the Last absolute shrinkage and selection operator (LASSO) Cox regression analysis by R package “glmnet” was performed to further select the most useful prognostic markers and the penalty regularization parameter lambda was chosen based on 10 cross-validations by R package “caret”. Through multiplying the expression level of a gene by its corresponding Cox regression coefficient, the risk score for each patient was calculated using the following formula:


$$\text{LRG\_score =}\;\sum_{i=1}^{n}Coefi\times Xi$$

The Coefi and xi represent the coefficient and expression levels of the corresponding gene, respectively. Based on the median risk score, all samples were separated into two categories: low-risk (LRG_score< median value) and high-risk (LRG_score > median value) groups. The differences in OS between the different risk groups were compared with Kaplan-Meier analysis. The time-dependent ROC curve was generated with the “survivalROC” R package, evaluating the accuracy of the prognostic LRG_score. Finally, the “rms” package was adopted to predict 1-, 3-, and 5-year survival by constructing a nomogram. The accuracy of the nomogram was evaluated by calibration, ROC, and decision curves.

### Integrated analysis of the LRG_score in LUAD

The abundance of 22 infiltrating immune cells in two LRG_score groups was quantified with CIBERSORT. The ESTIMATE algorithm was adopted to assess estimated score, stromal score, and immune score for each sample. The mutation of the genes in LUAD samples of TCGA database was analyzed with the “maftools” R package. The semi-inhibitory concentration (IC50) values of drugs were calculated with the “pRRophetic” package.

### qRT-PCR

The LUAD cell lines (A549, H1299, and HCC827), as well as the human normal bronchial epithelial cell line (BEAS2B) were kindly provided by the Cell Repository of the Chinese Academy of Sciences (Shanghai, China). All cell lines were cultured in RPMI-1640 medium containing 10% Fetal Bovine Serum (FBS), streptomycin (100 U/mL), and penicillin (100 U/mL) at 37°C in 5% CO_2_ atmosphere (14).

TRIzol^®^ (1 mL) was used to isolate total RNA from 20 pairs of clinical LUAD tumors and adjacent tissue (200 mg). Complementary DNA (cDNA) was created using reverse transcriptase from avian medulloblastoma virus and random primers according to TAKARA’s instructions. SYBR Premix Ex Taq II (Takara, Shiga, Japan) was adopted to perform qRT-PCR. Data were analyzed using 2 ^-ΔΔCT^ values. Primers for the genes are displayed in **Supplementary Table 1**. In addition, we provide the following information related to ethical approval: The Ethics Committee of the Second Hospital of Hebei Medical University approved the study protocol according to the guidelines of the Declaration of Helsinki.

### Statistical analysis

All the statistical analyses were made with the R software (v.4.0.1). The above section has described detailed statistical approaches for transcriptome data processing. A p-value less than 0.05 was of statistical significance.

## Results

### Expression and prognosis of LRGs in LUAD

**Figure 1:**
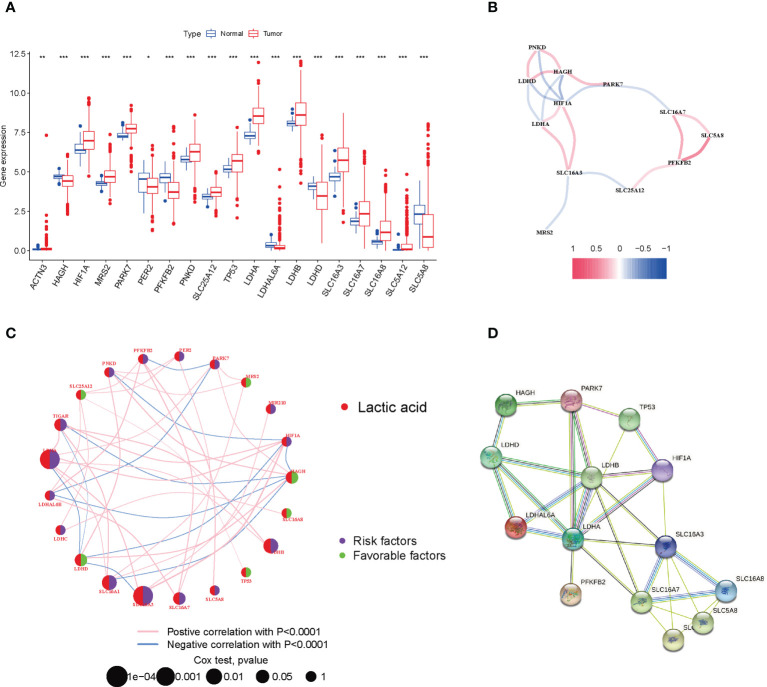
Expression and prognosis of LRGs in LUAD. **(A)** Expression difference of LRGs between normal and LUAD tissues. **(B)** The correlation of LRGs. **(C)** Mutual effects among LRGs in LUAD. The line linking to the LRGs stands for their interaction, with the line thickness suggesting the strength of the relationship between LRGs. Blue and pink stand for negative and positive correlations. **(D)** A PPI network of LRGs. *P < 0.05; **P < 0.01; ***P < 0.001.

We combined 59 normal tissues and 535 tumor tissues in the TCGA database to compare the expression levels of 24 LRGs. Among them, 13 genes (ACTN3, HIF1A, MRS2, PARK7, PNKD, SLC25A12, TP53, LDHA, LDHB, SLC16A3, SLC16A7, SLC16A8, and SLC5A12) were upregulated in tumor tissues, whereas 6 genes (HAGH, PER2, PFKFB2, LDHAL6A, LDHD, and SLC5A8) were upregulated in normal tissues (**Figure 1A**). Next, the correlation algorithm was adopted to analyze the expression of LRGs and drew a correlation network diagram to observe the co-expression relationship between LRGs (P< 0.001, R > 0.3; **Figure 1B**).

For fully understanding the expression pattern of LRGs in tumorigenesis, we showed the prognostic value of 24 LRGs in LUAD patients by univariate Cox regression and Kaplan-Meier analysis (**Supplementary Table 2**), and selected P< 0.05 as the screening threshold, six of which (LDHA, SLC16A3, SLC16A1, HAGH, LDHB, and LDHD) were identified as independent predictors. The integrated landscape of LRG interactions, regulator associations, and their prognostic value in patients with LUAD patients was illustrated in a lactic acid metabolism network (**Figure 1C**). To investigate the interaction between 24 LRGs, the PPI network was built with the online tool of the string website. Relatively close associations were found among the 14 LRGs (**Figure 1D**).

### Identification of lactic acid metabolism subtypes in LUAD

**Figure 2:**
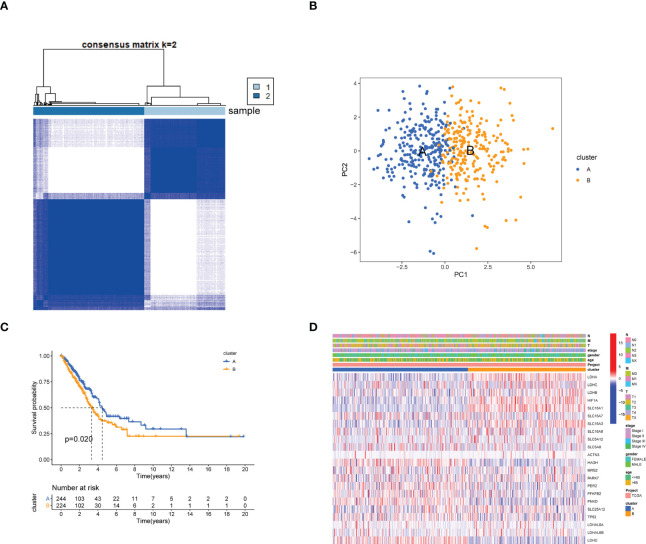
Identification of lactate metabolism subtypes in LUAD. **(A)** Consensus matrix heatmap defining two clusters (cluster 1=subtype A, cluster 2=subtype B, Samples are in columns, clustered iteration rows, and colors in cells indicate consensus values) (k = 2) and their correlation region. **(B)** PCA analysis showing a remarkable difference in transcriptomes between the two subtypes. **(C)** Kaplan-Meier survival curve in two subtypes. **(D)** Differences in clinicopathologic features and expression levels of LRGs between the two distinct subtypes.

To further explore the associations between the expression of the 24 LRGs and LUAD molecular subtypes, a consensus clustering analysis was conducted. When k = 2, the highest intragroup correlations and lowest intergroup correlations were found, suggesting that patients with LUAD could fall into two subtypes (cluster 1=subtype A, cluster 2=subtype B) (**Figure 2A**). PCA analysis revealed significant differences in the lactic acid metabolism transcription profiles between the two subtypes (**Figure 2B**). Furthermore, OS analysis displayed that LUAD patients with subtype A had a better prognosis compared with subtype B (P = 0.020, **Figure 2C**). Heatmap showed significant differences in clinicopathological characteristics between different subtypes (**Figure 2D**).

### Features of the TME in distinct subtypes

**Figure 3:**
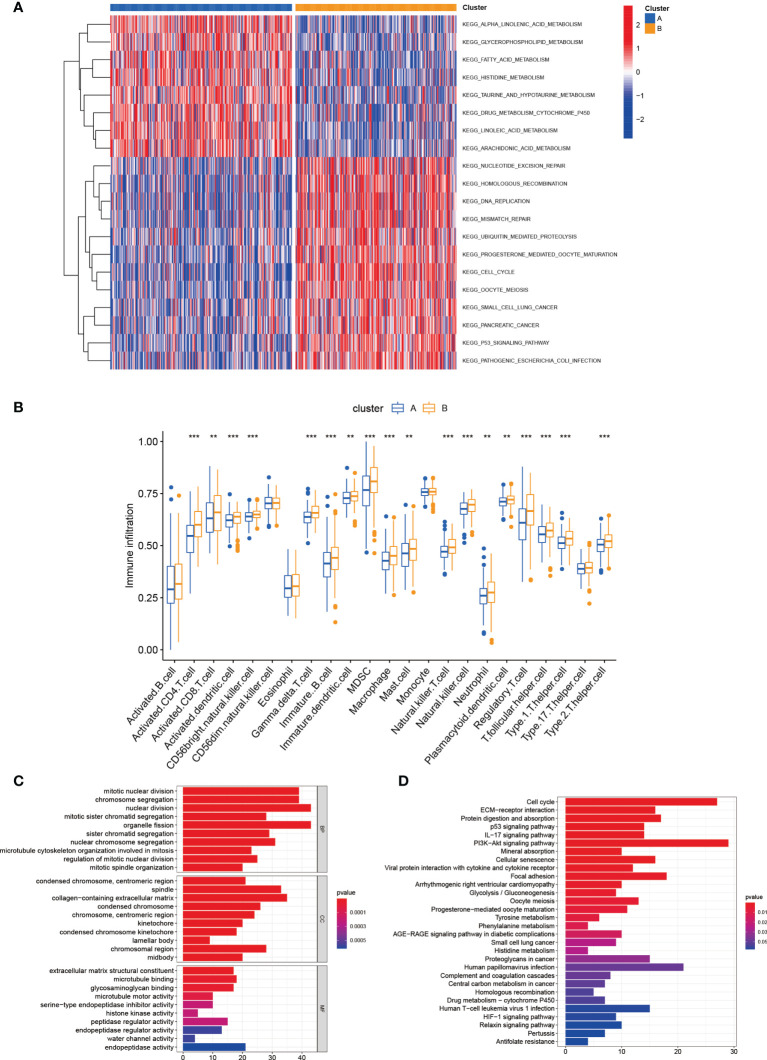
Correlations of tumor immune cell microenvironments and two LUAD subtypes. **(A)** GSVA of biological pathways between two distinct subtypes. **(B)** Abundance of 23 infiltrating immune cell types in two distinct subtypes. **(C, D)** GO and KEGG enrichment analyses of DEGs among two lactate metabolism subtypes. **P < 0.01; ***P < 0.001.

As indicated from the GSVA enrichment analysis, the enrichment of subtype A was found in metabolism-associated pathways, including alpha linolenic acid metabolism, glycerophospholipid metabolism, linoleic acid metabolism, fatty acid metabolism, histidine metabolism, drug metabolism cytochrome P450, taurine and hypotaurine metabolism, and arachidonic acid metabolism pathways (**Figure 3A**). Subsequently, the CIBERSORT algorithm was used to study immune infiltration in the TME between two different subtypes. The outcomes displayed that 18 of 23 immune cell infiltrations were greatly different between two subtypes, and subtype B had higher levels of immune cells infiltration (**Figure 3B**). For identifying the main biological characteristics and cell functional pathways of every lactate metabolism pattern, we identified 420 lactic acid metabolism subtype-related DEGs and made enrichment analysis. GO and KEGG analysis revealed the great enrichment of these lactic acid metabolism subtype-related genes in biological processes related to cancer (**Figures 3C, D**).

### Construction of lactic acid metabolism-related prognostic signature for LUAD patients in TCGA database

**Figure 4:**
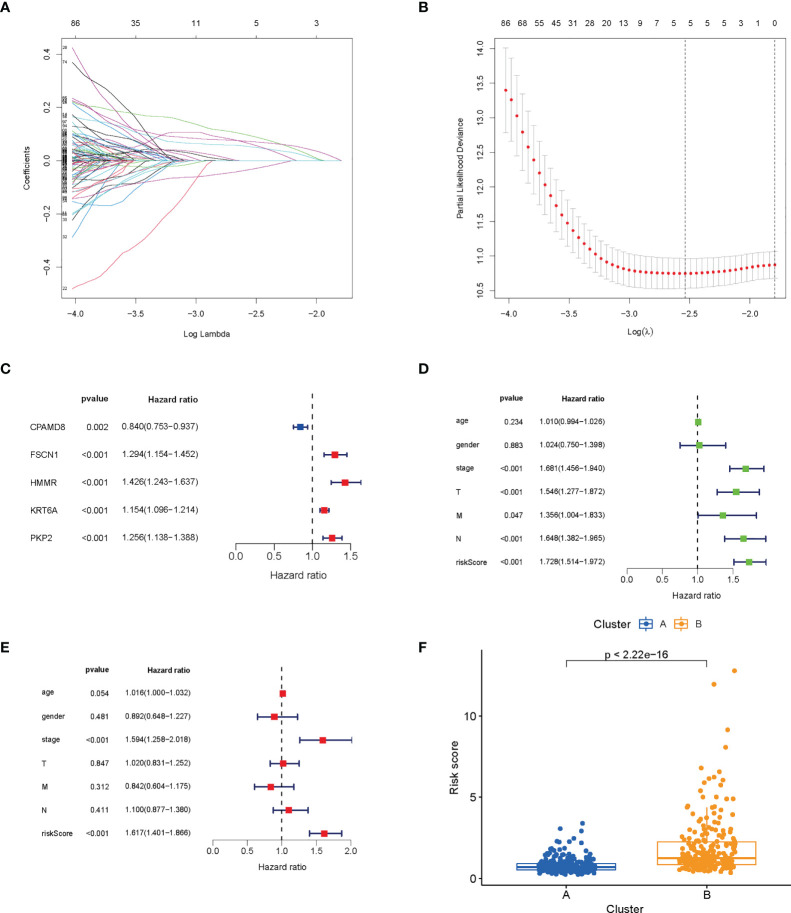
Construction and verification of prognosis LRG_score. **(A, B)** The LASSO regression analysis. **(C)** Forest plot of multivariate Cox regression analysis for prognostic genes. **(D, E)** Univariate and multivariate analyses showed the prognostic value of the LRG_score. **(F)** Differences in LRG_score between lactate metabolism subtypes.

First, we performed LASSO and multivariate Cox analyses for 420 lactic acid metabolism subtype-related DEGs for further selecting optimum prognostic signature. 5 genes remained on basis of the minimum partial likelihood deviance (**Figures 4A-C**). The LRG_score was constructed below: Risk score = (-0.1267 × CPAMD8 expression) + (0.1672 × HMMR expression) + (0.1268 × FSCN1 expression) + (0.1137 × PKP2 expression) + (0.0706 × KRT6A expression). Next, we performed COX analysis combining clinical characteristics such as age, gender, stage, T, M and N. Univariate and multivariate Cox regression analysis indicated that LRG_score can be adopted as a prognostic factor independent of clinical characteristics (**Figures 4D, E**). We also found a great diversity in LRG_score between two lactic acid metabolism subtypes (**Figure 4F**).

**Figure 5:**
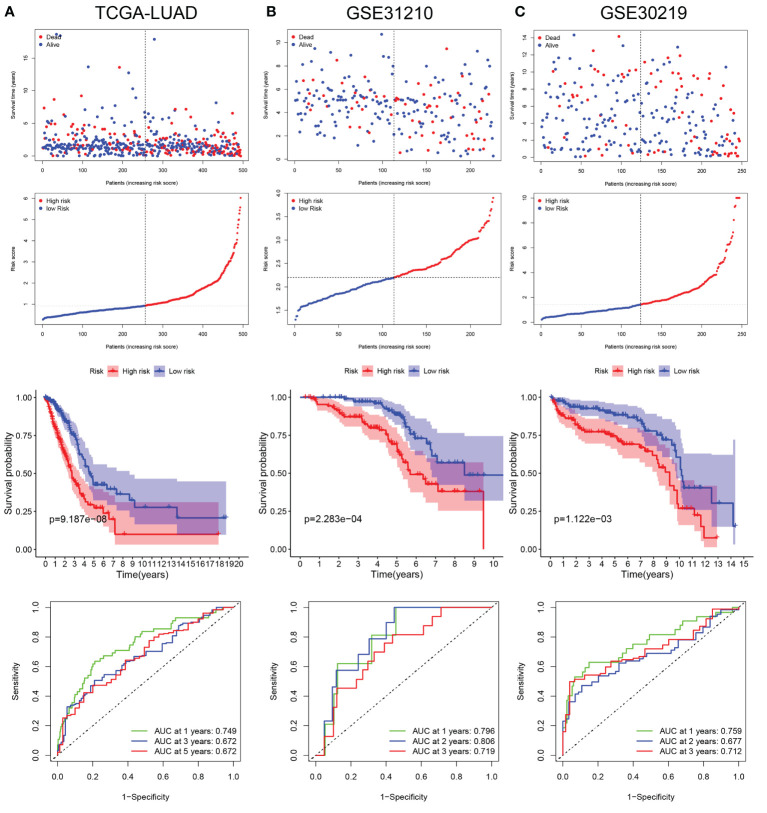
Validation of LRG_score in training set and validation sets. **(A)** The ranked dot plot indicates the LRG_score distribution, scatter plot presenting the patients’ survival status, KM survival curves, and area under the curve (AUC) value in training set (TCGA-LUAD). **(B)** The ranked dot plot indicates the LRG_score distribution, scatter plot presenting the patients’ survival status, KM survival curves, and area under the curve (AUC) value in validation set (GSE31210). **(C)** The ranked dot plot indicates the LRG_score distribution, scatter plot presenting the patients’ survival status, KM survival curves, and area under the curve (AUC) value in validation set (GSE30219).

LUAD patients in the TCGA database were divided into low- and high-risk groups according to the median risk score. The risk score plot and living status indicated that the low-risk group had better survival status and longer survival time. The K-M analysis suggested that the survival outcome of the low-risk group was significantly better. The ROC curve revealed that our prognostic signature exhibited good sensitivity and specificity in predicting LUAD patient OS (**Figure 5A**).

### Validation of lactic acid metabolism-related prognostic signature in two independent external cohorts

To further verify the universality of the constructed prognostic signature from the training cohort, two independent cohorts (GSE31210 and GSE30219) were introduced as validation groups. The LRG_score of each patient in these two validation cohorts was computed using the same formula as that for the TCGA cohort. The distribution plot of the LRG_score and survival status showed that the number of LUAD patients with dead status was gradually promoted with the increase of LRG_score in GSE31210 and GSE30219. Consistent with the training set, the high-risk group suffered poorer survival status than the low-risk group in validation sets. In addition, the AUC values of 1 year, 2 years, and 3 years for ROC analysis were 0.796, 0.806, and 0.719, respectively, in the GSE31210 cohort";"the AUC value of 1 year, 2 years, and 3 years were 0.759, 0.677, and 0.712, respectively, in the GSE30219 cohort (**Figures 5B, C**).

### Construction of the clinical nomogram

**Figure 6:**
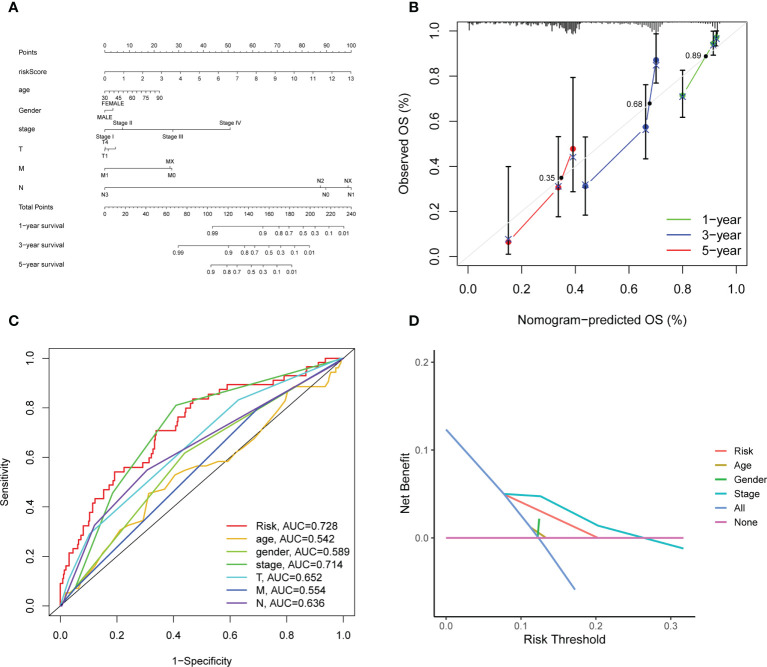
Construction and evaluation of the novel nomogram. **(A)** The nomogram for predicting the survival probability of LUAD patients. **(B)** The calibration plots of the nomogram for predicting OS probability for 1-, 3-, and 5-years. The intersection point of calibration curve of OS were 0.35 (1 year), 0.68 (3 years), 0.89 (5 years). **(C)** ROC analysis of the nomogram. **(D)** DCA of the nomogram.

Considering that the formula of risk signature is complicated and the nomogram can be applied visually to clinical work, we constructed a nomogram to predict 1-year, 3-year and 5-year OS of LUAD patients (**Figure 6A**). Furthermore, the calibration curve showed that its predicted curve was close to the true curve of LUAD patients, which indicates that the predicted survival rate is closely related to the actual rates at 1, 3, and 5 years (**Figure 6B**). The AUC of the ROC curves further confirmed that the predictive value of the nomogram was superior to any single prognostic factor (**Figure 6C**). Subsequently, DCA elicited that the nomogram obtained the best clinical benefit compared with the single prognostic factor (**Figure 6D**). The above results determined that the nomogram is suitable for clinically predicting the prognosis of LUAD patients.

### TME analysis

**Figure 7:**
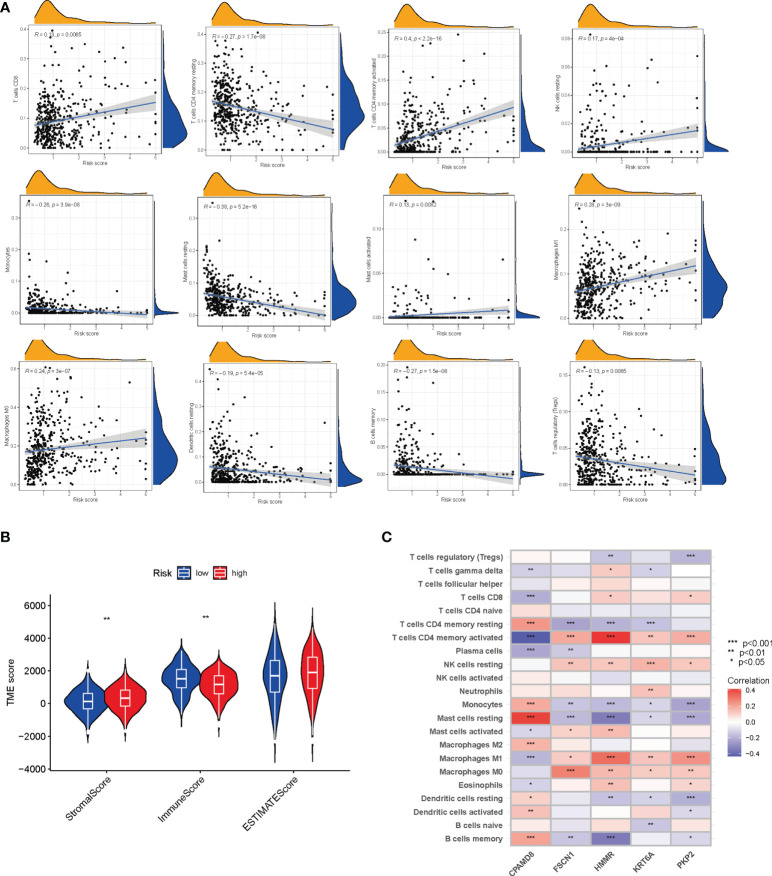
Evaluation of the TME between the two groups. **(A)** Correlations between LRG_score and immune cell types. **(B)** Correlations between LRG_score and both immune, estimate and stromal scores. **(C)** Correlations between the abundance of immune cells and five genes in the signature. *P < 0.05; **P < 0.01; ***P < 0.001.

**Figure 7A** showed the correlation between the LRG_score and immune cell infiltration. We found the positive correlation between the LRG_score and M0 macrophages, activated mast cells, CD4 + T cells, CD8 + T cells, M1 macrophages, and resting NK cells and negatively related to memory B cells, monocytes, resting dendritic cells, resting memory CD4 + T cells, resting mast cells, and T cells regulatory (Tregs). Patients with a low LRG_score presented a higher level of immune score and a lower stromal score than those with a high LRG_score (**Figure 7B**). Furthermore, the correlation of the 5 signature-associated genes and immune cell infiltration was also explored. We observed that 5 signature-related genes were geatly related to most immune cells (**Figure 7C**).

### Mutation, stemness and drug sensitivity analysis

**Figure 8:**
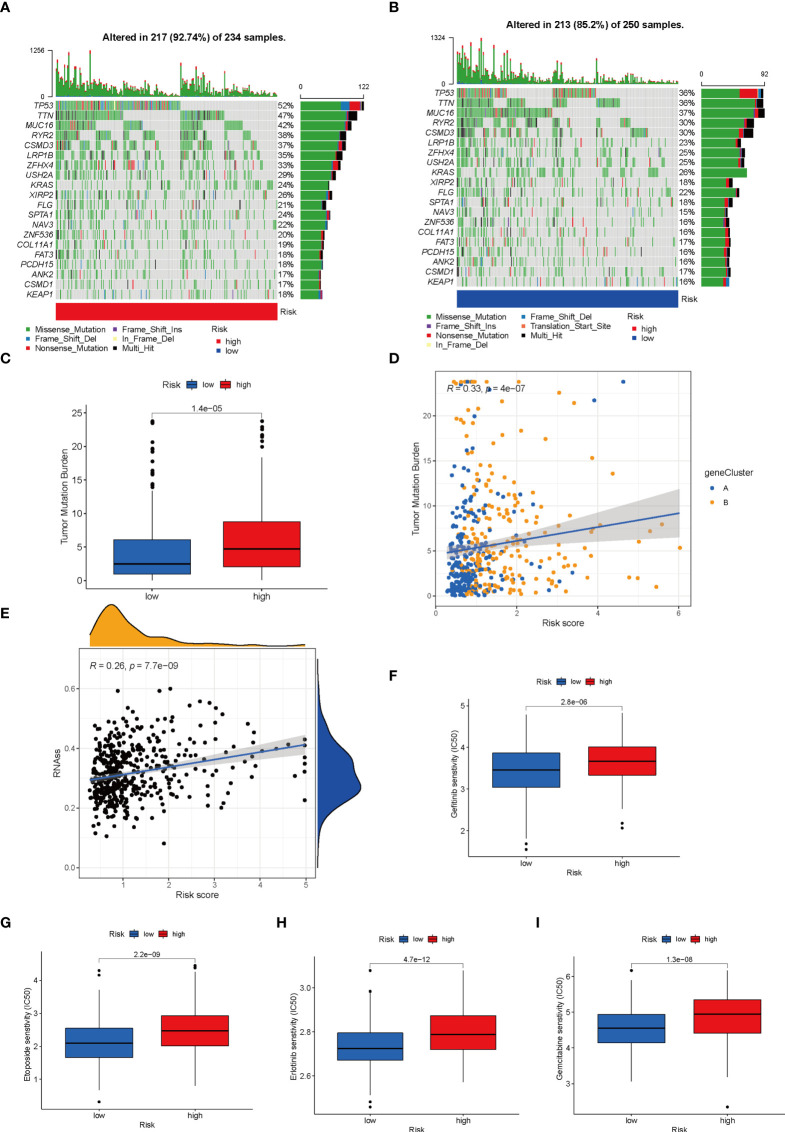
Comprehensive analysis of the LRG_score in LUAD. **(A, B)** The waterfall plot of somatic mutation features established with high and low LRG_scores. **(C)** TMB in different LRG_score groups. **(D)** Spearman correlation analysis of the LRG_score and TMB. **(E)** Relationships between LRG_score and CSC index. **(F-I)** Relationships between LRG_score and drug susceptibility.

Given that genetic mutations are key factors in tumorigenesis, we explored the situation of somatic mutations between two LRG_score groups. The relevant results are presented in the **Figures 8A, B**, and the somatic mutation rate of the high LRG_score group reached 92.74% (217 of the 234 samples), mainly the missense mutation. Of these, TP53 showed the highest frequency of mutations (52%). The mutation rate in the low LRG_score group was 85.2% (213 of the 250 samples), primarily the missense mutation. Of these, MUC16 showed the highest frequency of mutations (37%). It has been suggested that TMB can be used as a marker to identify patients with cancer who might benefit from immunotherapy and forecast the curative effect of immune checkpoint inhibitors. **Figure 8C** showed the difference in TMB in the high- and low-LRG_score groups. Moreover, the correlation analysis showed that the LRG_score was positively correlated with the TMB (**Figure 8D**). It was also investigated the association between the LRG_score and the CSC index. It was found that LRG_score had a positive correlation with CSC index, suggesting that LUAD cells with higher LRG_score had more distinct stem cell properties and a lower extent of cell differentiation (**Figure 8E**). Next, we determined the association between anticancer drug sensitivity and LRG_score with the Genomics of Drug Sensitivity in Cancer (GDSC) database. As shown in **Figures 8F-I**, gefitinib, etoposide, erlotinib, and gemcitabine had a lower IC50 value in low LRG_score group, indicating that these drugs may display better responses in treating the low LRG_score LUAD patients.

### Prediction of immunotherapy response

**Figure 9:**
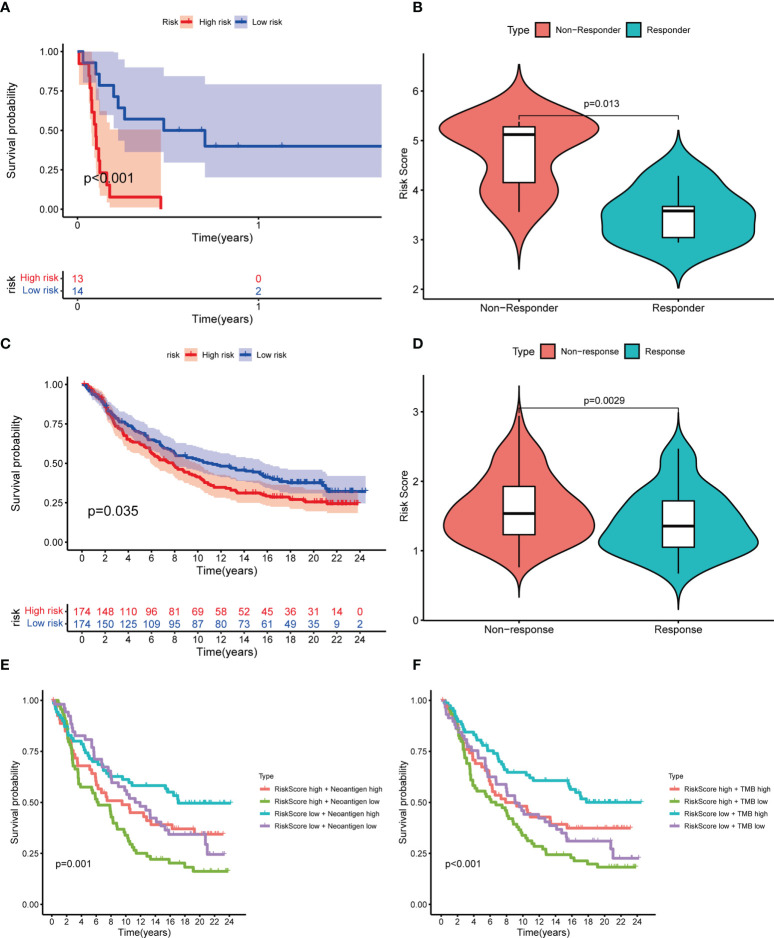
Prediction of response to immunotherapy. **(A)** Kaplan-Meier curves showing the poorer OS in NSCLC patients receiving anti-PD1 therapy of the high LRG_score group in GSE135222; **(B)** Violin plots showing significantly decreased LRG_score in patients responding to immunotherapy in GSE126044; **(C)** Kaplan-Meier curves for the high LRG_score and low LRG_score groups in the IMvigor 210 cohort;**(D)** Violin plots showing the significant difference in the LRG_score between patients responsive and irresponsive to immunotherapy in the IMvigor 210 cohort; **(E, F)** Kaplan-Meier curves for neoantigens **(E)** and TMB **(F)** between high and low LRG_score group in the IMvigor 210 cohort.

As mentioned above, there may be an association between LRG_score and immunotherapy response. Using the GSE135222 dataset of NSCLC patients receiving anti-PD-1 therapy, we found that those with high LRG_score had shorter survival (p< 0.001) (**Figure 9A**). Meanwhile, in the GSE126044 dataset, a reduction in the LRG_score was observed in patients who responded to immunotherapy (p=0.013) (**Figure 9B**). In addition, we used the IMvigor210 dataset to validate the results. Patients with a high LRG_score had a worse survival result (**Figure 9C**), and patients who had anti-PD-L1 treatment responses had low LRG_score (p=0.0029) (**Figure 9D**). Each patient’s neoantigens and TMB data are included in the IMvigor210 cohort. To understand why patients with low LRG_score are more likely to receive immunotherapy, we evaluated the survival between LRG_score, neoantigens, and TMB. In **Figure 9E, F**, we found that high levels of TMB and neoantigens were associated with the best survival expectations in the low LRG_score group. Taken together, the data suggest that patients with low LRG_score may also benefit more from immunotherapy and have better survival outcomes.

### Validation of core LRGs

**Figure 10:**
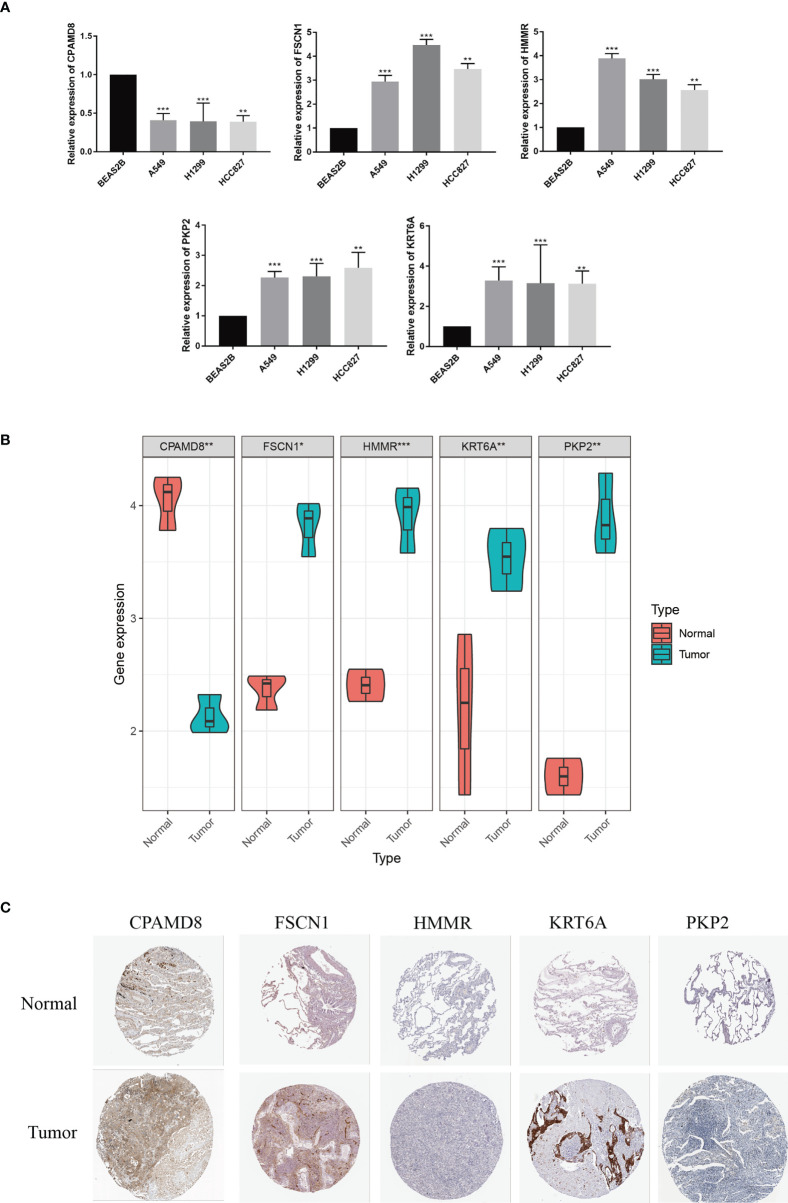
Validation of the expression patterns of 5 signature genes.The expression levels of five LRGs in the prognostic signature in normal bronchial epithelium cells and LUAD cells. **(B)** The expression levels of five LRGs in LUAD and adjacent normal tissues were examined by qRT-PCR. **(C)** HPA database showing the expression of signature genes in LUAD tissues compared with normal tissues. *P < 0.05; **P < 0.01; ***P < 0.001.

The expression level of the five signature-related genes in three LUAD cell lines (A549, H1299, and HCC827) and a normal bronchial epithelium cell line (BEAS2B) were validated. The outcomes suggested that FSCN1, HMMR, PKP2, and KRT6A showed greatly increased expression level in LUAD cells by comparing with normal bronchial epithelium cells, whereas CPAMD8 was downregulated in LUAD cells (**Figure 10A**). The same outcomes were detected in tumor tissue and matched adjacent normal tissue (**Figure 10B**). Second, in IHC sections in the HPA database, the protein expression of these genes in most tumour samples was higher than that in normal bronchial epithelium, and the staining was more intense (**Figure 10C**). To be collective, these ooutcomes further verified the stability and reliability of the LRG_score.

## Discussion

As we all know, this is the first study to explore the association between LRGs and LUAD. In the present study, we first systematically studied the expression and prognosis of 24 LRGs in patients with LUAD. Two distinct molecular subtypes were identified based on 24 LRGs. By comparing with patients with subtype B, patients with subtype A had better OS. According to GSVA enrichment analysis, subtype A was significantly enriched in metabolism-related pathways. The level of immune cell infiltration also different greatly between the two subtypes. Subsequently, a prognostic signature based on 5 OS-related LRGs was generated using Lasso Cox hazards regression analysis in TCGA dataset and was validated in two external cohorts. Univariate and multivariate Cox regression analysis indicated that LRG_score can be adopted as a prognostic factor independent of clinical characteristics. Patients were separated into two groups based on the median value of risk score: high LRG_score and low LRG_score groups. Patients in the low- and high-LRG_score groups showed significant differences in prognosis, TME, mutations, TMB, CSC index, and drug sensitivity. Based on the IC50 values, gefitinib, etoposide, erlotinib, and gemcitabine showed a better response in the treatment of the low LRG_score LUAD patients. Finally, by integrating the LRG_score and clinicopathological parameters, a quantitative nomogram was set up. The results of ROC and calibration curves displayed that the nomogram had great prognostic predictive performance.

HMMR is a multifunctional protein that regulates cancer progression. Elevated HMMR expression is a feature of most cancers and is also an indicator of poor prognosis in many cancers (15). In addition, its ability to promote inflammation and fibrosis exacerbates the progression of cancer (16, 17). As an action-bundling protein that cross links actin filaments, FSCN1 promotes cell migration and invasion by generating stable filopodia and invadopodia (18, 19). Some researches have also documented that targeted inhibition of FSCN1/actin bundling blocks tumor cell migration and metastasis (20–22). In addition, FSCN1 regulates tumor cell migration, invasion and metastasis by participating in key oncogenic pathways such as EMT, PI3K/AKT, Wnt/β-catenin, and MAPK (23–25). The wide expression of PKP2 is observed in epithelial cells (26). Wu et al. disclosed that PKP2 could drive LUAD progression by enhancing local adhesion and epithelial-mesenchymal transition (27). Yang et al. (28) reported that KRT6A was overexpressed in LUAD and promoted the proliferation and migratory ability of LUAD cells through EMT and CSC transformation. A recent study showed that KRT6A promotes growth and invasion of lung cancer cells by regulating glucose-6-phosphate dehydrogenase expression through MYC (29).

This study revealed an association between LRG_score and immune cell infiltration. We found that the LRG_score was positively related to M0 macrophages, activated mast cells, CD4 + T cells, CD8 + T cells, M1 macrophages, and resting NK cells and negatively related to memory B cells, monocytes, resting dendritic cells, resting memory CD4 + T cells, resting mast cells, and T cells regulatory (Tregs). These results indicate that lactic acid metabolism partially regulates the TME. And the LRG_score may aid in the discovery of the regulatory mechanism of tumor immunity and provide new insights for future TME investigations. Patients with low LRG_score presented higher immune score and lower stromal score than those with high LRG_score. In addition, our study indicated that 5 signature-related genes were significantly correlated with most immune cells, suggesting that the LRG_score could be used as a new immunological indicator in the treatment of LUAD. These outcomes offer a reference for further researches on the mechanisms of LRGs in LUAD.

TMB is significant predictive biomarkers for cancer immunotherapy (30). Considering the importance of TMB in tumour immunotherapy, we analysed the differences in TMB between the two groups. According to the outcomes, the high LRG_score group had higher TMB scores. Hence, patients with high LRG_score might benefit more from immunotherapy compared to patients with low LRG_score. We also found that LRG_score was positively related to the CSC indicator, suggesting that LUAD cells with higher LRG_score had more distinct stem cell nature and a lower extent of cell differentiation. We also found that gefitinib, etoposide, erlotinib, and gemcitabine showed a better response in the treatment of the low LRG_score LUAD patients, suggesting that prognostic signature is a powerful predictive tool for drug sensitivity and could promote personalized cancer treatments in the future.

Three more independent cohorts (GSE126044: anti-PD-1, GSE135222: anti-PD-1, and IMvigor210: anti-PD-L1) were analyzed, and the results showed that patients in the low LRG_score category were more responsive to anti-PD-1/PD-L1 medication and had longer survival times. This provides more convincing evidence. When the LRG_score was high, NSCLC patients receiving anti-PD-1/PD-L1 medication had a worse survival outcome. This further demonstrated that patients with low LRG_score may also benefit more from immunotherapy and have better survival outcomes. Neoantigens and TMB information for each patient are also included in the IMvigor210 cohort. As a consequence, we analyzed the relationship between TMB, neoantigens, and risk score. We discovered that high levels of TMB and neoantigen were associated with the best survival expectations in the low LRG_score group. Collectively, these data further support that the LRG_score can be used to inform treatment decision utilizing data from randomized clinical studies that can inform on its prognostic or predictive value.

## Conclusion

In conclusion, the lactate metabolism patterns were evaluated with the lactic acid prognostic signature in an integrated way. Patients’ clinicopathological features such as molecular subtypes, mutation status, TMB status, and CSC index can be characterized with the LRG_score. In addition, the LRG_score is related to patients’ prognosis and can also forecast the sensitivity to chemotherapy and immunotherapy. This signature may serve as a predictor of prognosis and immunotherapy for LUAD in the future.

## Data availability statement

The original contributions presented in the study are included in the article/**Supplementary Material**. Further inquiries can be directed to the corresponding author.

## Ethics statement

The studies involving human participants were reviewed and approved by the Ethics Committee of The Second Hospital of Hebei Medical University. The patients/participants provided their written informed consent to participate in this study.

## Author contributions

FZ and S-JL conceived and designed the study. FZ and ZW wrote the manuscript and participated in data analysis. ZL and S-QL participated in discussion and language editing. S-JL reviewed the manuscript. All authors contributed to the article and approved the submitted version.

## Funding

This study was supported by Science and Technology Program of Hebei (223777106D and 19270118D).

## Conflict of interests

The authors declare that the research was conducted in the absence of any commercial or financial relationships that could be construed as a potential conflict of interest.

## Publisher’s note

All claims expressed in this article are solely those of the authors and do not necessarily represent those of their affiliated organizations, or those of the publisher, the editors and the reviewers. Any product that may be evaluated in this article, or claim that may be made by its manufacturer, is not guaranteed or endorsed by the publisher.
