# Supplementary material for: Identifying a lactic acid metabolism-related gene signature contributes to predicting prognosis, immunotherapy efficacy, and tumor microenvironment of lung adenocarcinoma
Source: Front Immunol. 2022 Oct 7;13:980508. doi: 10.3389/fimmu.2022.980508 (PMC9585198; doi:10.3389/fimmu.2022.980508)
Supplement: Supplementary file 1 [file Table_1.docx]

**Table S1: The primer sequences used in the present study.**

| **lncRNA** | **Forward primer (5′-3′)** | **Reverse primer (5′-3′)** |
| --- | --- | --- |
| FSCN1 | 5'-GGTCAACATCTACAGCGTCAC-3' | 5'-GCGCCTACAACATCAAAGACT-3' |
| HMMR | 5'-AACAAGCTGAAAGGCTGGTCA-3' | 5'-GGGTATGAGCAGCACTACTTTT-3' |
| PKP2 | 5ʹ-AGATTACCAGCCAGATGACA-3ʹ | 5ʹ-ATGCCACAGCCACTCCAC-3ʹ |
| KRT6A | 5'-TCACCGTCAACCAGAGTCTC-3' | 5'-GAACCTTGTTCTGCTGTCCC-3' |
| CPAMD8 | 5'-GATGGGAAGTCCGTCAGACC-3' | 5'-TGGACTCTCTCGTTGGGACA-3' |

。
