# Supplementary material for: Identifying a lactic acid metabolism-related gene signature contributes to predicting prognosis, immunotherapy efficacy, and tumor microenvironment of lung adenocarcinoma
Source: Front Immunol. 2022 Oct 7;13:980508. doi: 10.3389/fimmu.2022.980508 (PMC9585198; doi:10.3389/fimmu.2022.980508)
Supplement: Supplementary file 2 [file Table_2.docx]

**Table S2: Cox and KM analysis of lactic acid metabolism-related genes**

| gene | HR | HR.95L | HR.95H | pvalue | km |
| --- | --- | --- | --- | --- | --- |
| ACTN3 | 0.847685 | 0.387564 | 1.854067 | 0.678997 | 0.121001 |
| HAGH | 0.700836 | 0.526324 | 0.933211 | 0.014971 | 0.004438 |
| HIF1A | 1.173477 | 0.969003 | 1.421098 | 0.1015 | 0.028265 |
| MRS2 | 0.951088 | 0.745999 | 1.21256 | 0.685714 | 0.043621 |
| PARK7 | 1.404471 | 0.99093 | 1.990592 | 0.056293 | 0.013472 |
| PER2 | 1.129672 | 0.917032 | 1.391619 | 0.251822 | 0.067553 |
| PFKFB2 | 0.938539 | 0.757651 | 1.162614 | 0.561465 | 0.041311 |
| PNKD | 0.969053 | 0.784871 | 1.196456 | 0.770067 | 0.096652 |
| SLC25A12 | 0.960949 | 0.679129 | 1.359716 | 0.822039 | 0.13456 |
| TP53 | 0.945596 | 0.793802 | 1.126418 | 0.530939 | 0.060133 |
| LDHA | 1.734025 | 1.414075 | 2.126368 | 1.23E-07 | 3.08E-08 |
| LDHAL6A | 0.914734 | 0.645325 | 1.296616 | 0.6166 | 0.206246 |
| LDHAL6B | 1.423736 | 0.85395 | 2.373703 | 0.175547 | 0.005476 |
| LDHB | 1.204254 | 1.034658 | 1.401649 | 0.0164 | 3.02E-05 |
| LDHC | 1.057231 | 0.892837 | 1.251893 | 0.518659 | 0.088113 |
| LDHD | 0.871777 | 0.771649 | 0.984897 | 0.027493 | 0.010219 |
| SLC16A1 | 1.219386 | 1.104419 | 1.34632 | 8.65E-05 | 3.79E-07 |
| SLC16A3 | 1.399293 | 1.189348 | 1.646298 | 5.11E-05 | 2.03E-07 |
| SLC16A7 | 1.090489 | 0.945866 | 1.257225 | 0.232747 | 0.055253 |
| SLC16A8 | 0.966825 | 0.823448 | 1.135165 | 0.680373 | 0.202615 |
| SLC5A12 | 1.078862 | 0.870729 | 1.336747 | 0.487597 | 0.059096 |
| SLC5A8 | 0.975359 | 0.877904 | 1.083632 | 0.642262 | 0.006455 |
